# Detection of *Babesia odocoilei* in *Ixodes scapularis* Ticks Collected in Southern Ontario, Canada

**DOI:** 10.3390/pathogens10030327

**Published:** 2021-03-10

**Authors:** John D. Scott, Emily L. Pascoe, Muhammad S. Sajid, Janet E. Foley

**Affiliations:** 1 Department of Medicine and Epidemiology, School of Veterinary Medicine, University of California Davis, Davis, CA 95616, USA; elpascoe@gmail.com (E.L.P.); jefoley@ucdavis.edu (J.E.F.); 2 Faculty of Veterinary Science, University of Agriculture, Faisalabad 38040, Pakistan; drsohailuaf@uaf.edu.pk

**Keywords:** *Babesia odocoilei*, piroplasm, babesiosis, ticks, *Ixodes scapularis*, parasitism, domestic cats, domestic dogs, Canada

## Abstract

Tick-borne zoonotic diseases have an economic and societal impact on the well-being of people worldwide. In the present study, a high frequency of *Babesia odocoilei*, a red blood cell parasite, was observed in the Huronia area of Ontario, Canada. Notably, 71% (15/21) blacklegged ticks, *Ixodes scapularis*, collected from canine and feline hosts were infected with *B. odocoilei*. Consistent with U.S. studies, 12.5% (4/32) of questing *I. scapularis* adults collected by flagging in various parts of southwestern Ontario were positive for *B. odocoilei*. Our data show that all *B. odocoilei* strains in the present study have consistent genetic identity, and match type strains in the GenBank database. The high incidence of *B. odocoilei* in the Huronia area indicates that this babesial infection is established, and is cycling enzootically in the natural environment. Our data confirm that *B. odocoilei* has wide distribution in southern Ontario.

## 1. Introduction

Tick-borne zoonotic pathogens cause many diseases that have considerable medical, veterinary, and economic impact worldwide. In North America, *Babesia odocoilei* is a single-celled microorganism that belongs to the genus *Babesia* (Apicomplexa: Piroplasmida: Babesiiidae). This intraerythrocytic piroplasm infects terrestrial vertebrate hosts [1]. Clinical manifestations of babesiosis range from a silent infection to a fulminating malaria-like disease to severe hemolysis that can infrequently result in human death [2,3,4,5,6,7,8,9,10,11,12]. *Babesia* infection is normally transmitted to humans by a tick bite; however, this babesial hemoparasite can be passed from infected individuals to others via blood transfusion [13,14,15], organ transplantation [16], and maternal-fetal transmission [17,18]. *Babesia* that are pathogenic to humans include *B. crassa, B. duncani, B. divergens*, *B. microti, B. venatorum*, *Babesia divergens*-like MO-1, *Babesia* sp. KO1, *Babesia* sp. XXB/HangZhou, *Babesia* sp. TW1, and *Babesia* spp. CA1, CA3, and CA4 [7,19,20,21,22]. Around the globe, there are at least 111 valid *Babesia* species [1].

Members of certain biological families, such as cervids and bovids, are reservoir hosts of *B. odocoilei*. Both feral and captive white-tailed deer, *Odocoileus virginianus* (Mammalia: Cervidae), are reservoir-competent hosts of *B. odocoilei* [23,24,25]. As well, wapiti/elk (*Cervus elaphus canadensis*), reindeer (*Rangifer tarandus tarandus*), and caribou (*Rangifer tarandus caribou*) are native reservoirs of *B. odocoilei* [26].

Although it was initially believed that *B. odocoilei* was non-pathogenic [27,28,29,30], it is now recognized as a cause of cervid babesiosis, a disease that can be fatal for cervids, such as white-tailed deer, caribou and wapiti, particularly immunocompromised or excessively stressed individuals [25,26,31]. More recently, *B. odocoilei* has been detected in bovids, such as desert bighorn sheep (*Ovis canadensis nelsoni*), musk oxen (*Ovibos moschatus*), yak (*Bos grunniens*), and markhor goat (*Capra falconeri*), including areas outside of the range of *I. scapularis* [32,33].

The blacklegged tick, *Ixodes scapularis* (Acari: Ixodidae), is the principal vector of *B. odocoilei*. This ixodid tick species parasitizes at least 150 terrestrial vertebrates (avian, mammalian, reptilian), including humans, songbirds, and white-tailed deer [34,35]. Once a host-seeking *I. scapularis* tick becomes infected with *B. odocoilei* [25,36,37,38,39], this babesial infection can be sustained by transstadial passage (larva to nymph or nymph to adult) and by transovarial transmission (female to eggs to larvae). Blacklegged ticks can perpetuate *B. odocoilei* throughout all life stages [25,37,38,39]. East of the Rocky Mountains, *I. scapularis* closely coincides with the distribution of white-tailed deer [25,27]. The wide dispersal of *B. odocoilei*-infected ticks is facilitated by migratory songbirds, especially during northward spring migration [35,40,41].

The primary aim was to determine the distribution of *B. odocoilei* in questing and animal-derived *I. scapularis* ticks in southern Ontario.

## 2. Materials and Methods

### 2.1. Tick Collection

Questing ticks were collected by flagging low-lying vegetation in the southern part of southwestern Ontario within the Carolinian forest region from 24 to 26 April 2019. At a more northerly location in the Huronia area, veterinarians and technicians collected attached, engorged ticks from domestic dogs and domestic cats from 2 to 27 May 2019. These attached ticks were removed using superfine-tipped stainless steel forceps, and ticks from each host were stored in a tightly sealed microtube containing 94% ethanol. Each microtube was labelled with a tick identification number. A white, vinyl-backed flannel cloth attached to a telescoping, aluminum pole was employed to collect questing ticks. Blood-fed and questing ticks were identified to species by using microscopy and taxonomic keys [34,42]. 

### 2.2. DNA Extraction, PCR, and Sequencing

To extract DNA from unfed ticks, an ammonium hydroxide protocol (unfed ticks) [35], or the Qiagen DNeasy Blood and Tissue Kit (Qiagen, Valencia, CA, USA) following the manufacturer’s protocol for animal tissue was used. The resulting DNA was stored at −20 °C until PCR was performed. Amplification of the 18S rRNA gene of *Babesia* was performed as previously described using the BJ1 (5′-GTC-TTG-TAA-TTG-GAA-TGA-TGG-3′) and BN2 (5′-TAG-TTT-ATG-GTT-AGG-ACT-ACG-3′) primers [43]. Amplicons were visualized by UV transillumination on a 1% agarose gel containing GelStar nucleic acid stain (Lonza, Rockland, ME, USA), and those that were 400−500 nucleotides in length, were excised from the gel, and prepared for DNA sequencing to confirm *Babesia* presence and species using the QIA amp DNA Kit (Qiagen, Valencia, CA, USA). DNA sequencing was performed at the University of California Davis Sequencing facility using the Big Dye Terminator cycle sequencing kit (Applied Biosystems, Foster City, CA, USA) and PCR primers.

### 2.3. Phylogenetic Analysis

End-reading errors were removed from sequences and, when possible, ambiguous nucleotides were manually corrected. Sequences were compared to those published in GenBank using the BLAST database search program (https://blast.ncbi.nlm.nih (3 November 2020). The phylogenetic tree was constructed based on select published sequences of *Babesia* species (*B. odocoilei*, *B. bovis*, *B. conradae*, *B. divergens*, *B. duncani*, *B. canis canis*, *B. gibsoni*, *B. microti*, and *B. vulpes*) downloaded from GenBank. *Babesia bovis* was used as the outgroup species. All sequences were trimmed to the same length (445 nucleotides, including those absent in some species), and were aligned using the MUSCLE algorithm [44]. Phylogeny was resolved using the maximum likelihood method in MEGA 10.0.5 [45]. This general time reversible model facilitated gamma distribution and invariant sites (number of discrete categories equals five) was determined by jModeltest 2.1.10 [46]. Consensus was achieved by bootstrapping based on 1000 pseudoreplicate datasets generated from the original sequence alignments.

## 3. Results

### 3.1. Tick Collection

**Figure 1:**
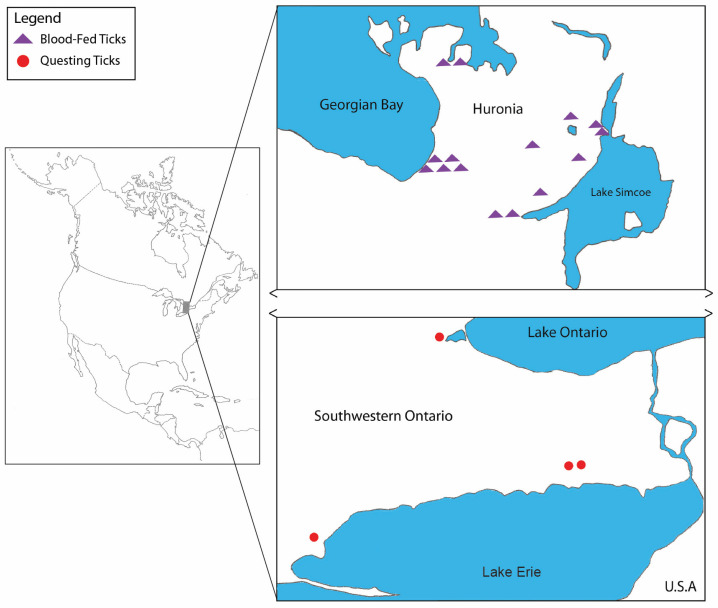
Map shows locations where *Ixodes scapularis* females were positive for *Babesia odocoilei* in southern Ontario, Canada. Purple triangles designate positive ticks collected from domestic dogs and domestic cats. Red dots represent positive ticks collected by flagging low-lying vegetation.

Between 24 April and 22 May 2019, a grand total of 53 *I. scapularis* adults (males, n = 13; females, n = 40) were collected from 14 locations in southern Ontario). These collections were conducted within two different forested areas (Great Lakes-St. Lawrence and Carolinian). Flagging was done in the Carolinian forest region, whereas canine- and feline-derived ticks were obtained from three veterinary clinics located in the Huronia area (Figure 1). Collections of *I. scapularis* comprised of 32 ticks (males, n = 13; females, n = 19) collected by flagging, and 21 females collected from 21 companion animals (domestic dogs, n =17; domestic cats, n = 4).

### 3.2. Babesia Detection

Of the 53 ticks, 35.8% (*n* = 19) tested positive for *Babesia* DNA (Table 1 and Table 2). The majority of positive ticks were from dogs (68.4%, *n* = 13), plus two from cats (10.5%), and four by flagging (21.1%; Table 2). All positive samples were confirmed to be *B. odocoilei* based on 99.72–100% similarity to sequences published in GenBank (Table 1). With the exception of three male ticks collected at flagging sites (Dundas, Turkey Point, Wainfleet bog), all *Babesia*-positive ticks were adult females (Table 1).

**Figure 2:**
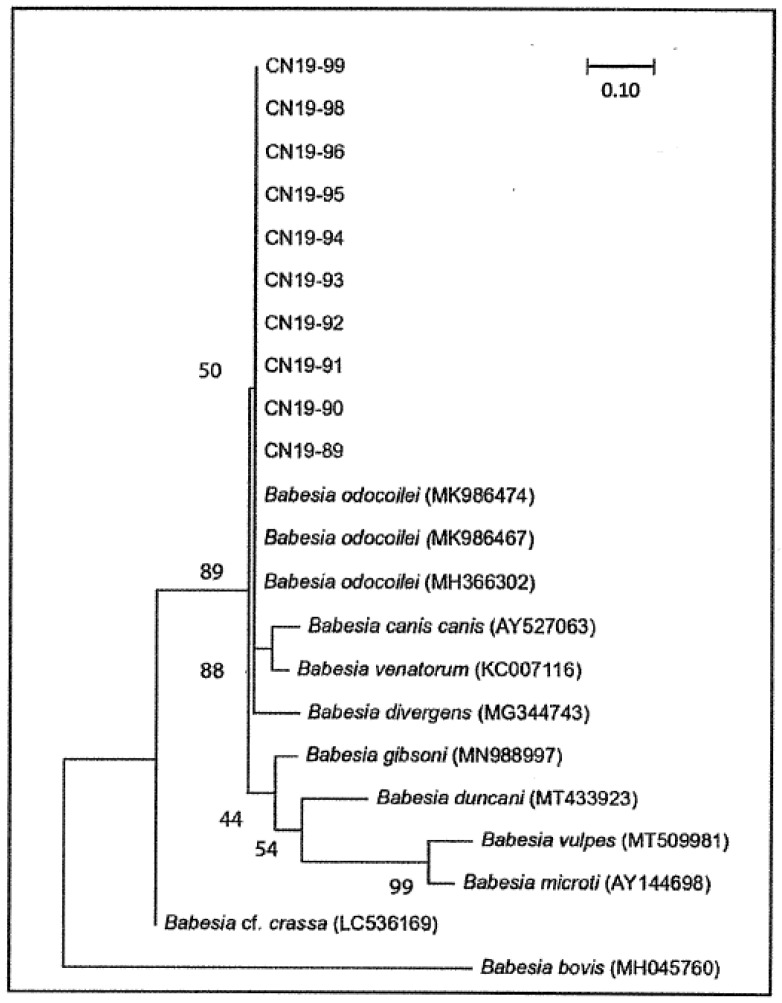
Maximum likelihood phylogenetic tree of 18S rRNA sequences from *Babesia* positive *Ixodes scapularis* ticks collected in southern Ontario in 2019. All sequences were trimmed to 445 nucleotides in length (including those absent to some species) and aligned using the MUSCLE algorithm. Phylogeny was resolved using a gamma distribution with invariant sites, and consensus was achieved by bootstrapping based on 1000 pseudoreplicate datasets generated from the original sequence alignments. Alphanumeric values in brackets denote published sequences. *Babesia bovis* is the outgroup species. The scale bar signifies the percentage of genetic variation along tree branches. Sequences from ticks are available in GenBank (accession numbers: MW182504−MW182513).

Between one and eight ticks (mean = 3.79) were collected from each location, and *Babesia*-positive ticks were collected from ten of the 14 sampled locations. The number of positive *I. scapularis* ticks collected at each positive location in both forested area was low (1–5), but prevalence ranged between 12.5–100% (mean = 65.06%; Table 1). Only ten of the 19 *B. odocoilei* sequences were of ample quality and length to perform phylogenetic analysis (CN19-89—CN19-99). These sequences were 100% similar to one another and to three reference strains of *B. odocoilei* downloaded from GenBank (Table 2, Figure 2).

## 4. Discussion

Here we report the presence of *Babesia* in *I. scapularis* ticks from ten of 14 sampled locations in Ontario, Canada, collected either by flagging low-lying vegetation or from domestic cats and dogs (Table 1). *Babesia* DNA was detected in 19 (35.8%) of 53 collected ticks. Our results are in concordance with previous studies conducted in the U.S.A. where 11−15% of the questing blacklegged tick adults collected from established populations were positive for *B. odocoilei* [47,48]. Although the gender of *I. scapularis* adults may seem disproportionate (Table 1), the ratio of males to females are balanced in nature genetically reflecting a 50:50 ratio. Based on deer tracks along trails, we observed that an *I. scapularis-O. virginianus* interface was present in each of the woodland and ecotone locations flagged. The high incidence of *B. odocoilei* in *I. scapularis* ticks in the Huronia area suggests that an epizootic, babesial infection is present in the local white-tailed deer population [23,24,25]. To our knowledge, this is the first time that *B. odocoilei* has been detected in ticks collected from dogs and cats in Canada. Researchers in Indiana reported one *B. odocoilei*-positive *I. scapularis* in 15 adults collected from a domestic dog [49]. Globally, certain other *Babesia* spp. (*B. canis* sensu stricto, *B. gibsoni*, *B. microti*, *B. vogeli*) can infect either cats or dogs [50]. In addition, *B. conradae* is associated with canine-feeding ticks and host dogs [51,52]. *Babesia* DNA, determined to be 97.8% similar to *B. odocoilei*, has been detected in ticks collected from dogs in Japan [53], whilst *B. vogeli* and *B. microti* have been detected in ticks associated with pet cats [54,55,56].

*Babesia odocoilei* can either be maintained in *I. scapularis* ticks by transstadial passage and/or transovarial transmission. All *B. odocoilei*-positive ticks in the present study were adults, and it is likely that they became infected during a previous developmental life stage while feeding on an infected reservoir host. As we did not draw blood from domestic cats and domestic dogs, we do not know if these companion animals play a role as reservoir hosts of *B. odocoilei.* This aspect warrants further investigation.

During the past half century, several tick-pathogen studies in North America have demonstrated that *B. odocoilei* has a wide distribution. In the U.S.A., the pathogen has been reported in ticks and vertebrate hosts as far north and east as Maine [47,49,57], as far south as Texas [58], and as far west as California [32]. The presence of *B. odocoilei* in California is notable because it has been detected in desert bighorn sheep, a non-cervid vertebrate host, which is outside the normal distribution of *I. scapularis* ticks [32]. Of biogeographical significance, *B. odocoilei* has been detected in the western blacklegged tick, *Ixodes pacificus*, and, thus, could be a vector infecting bovids in California. North of the Canada−U.S. border, *B. odocoilei* has been reported in Ontario [35,39,40,41], Quebec [35], and Saskatchewan [26]. Additionally, other epidemiological studies have reported *B. odocoilei* causing fatal outcomes in bovid species in captivity [32,33].

Ecologically, the number of *I. scapularis* adults in the spring is lower than the full complement of adults in the fall because the number of questing females goes down as they parasitize hosts, and remain in quiescence until spring to lay eggs. By spring, the number of adults is predictably lower. As *I. scapularis* females parasitize suitable hosts, the number of questing females decreases.

Of epidemiological significance, *B. microti* has also been detected in the Huronia area in the *Ixodes cookei* (groundhog tick) [39]. As *I. cookei* does not parasitize songbirds, *B. microti* is most likely established in the area. Therefore, people may become infected with this babesial piroplasm.

Songbirds play an important role in the wide dispersal of *B. odocoilei*-positive *I. scapularis* [39,40,41]. Therefore, people do not have to visit an endemic area to contract babesiosis. The symbiosis of blacklegged ticks and white-tailed deer in a sylvatic habitat provides the strategic components for a *B. odocoilei* endemic area. White-tailed deer are hosts of *I. scapularis*, especially males and female adults, and promote the propagation of this tick species. Currently, there is no risk map in Ontario for *Babesia*, so there is no way to see where these areas are located. Holding fast to the dogma that one must visit an endemic area might hamper the assessment and whereabouts of these areas by epidemiologists and healthcare providers. Any of the *B. odocoilei*-positive sites in southern Ontario may have been initiated by songbirds infested with *B. odocoilei*-infected *I. scapularis* larvae and nymphs. Migratory songbirds can transport bird-feeding ticks from as far south as equatorial South America [59,60,61,62,63,64,65,66,67], and potentially start a new foci of *I. scapularis* that is hundreds of kilometres from its original source [68,69,70,71]. These established populations of *I. scapularis* may unknowingly be infected with *B. odocoilei*.

Even though the pathogenicity in cats, dogs, and humans has not been clarified for *B. odocoilei*, this piroplasm is in the same clade/group as *Babesia divergens* and *Babesia venatorum* (Figure 2), both of which are pathogenic to humans [1,4]. In essence, healthcare providers must be vigilant to look for human babesosis in symptomatic patients, especially when bitten by blacklegged ticks.

## 5. Conclusions

We provide the first documentation of *B. odocoilei* in *I. scapularis* ticks collected from domestic dogs and cats in Canada. Of 21 ticks collected from domestic cats and domestic dogs, 71% were confirm positive for *B. odocoilei*. *Babesia odocoilei*-positive *I. scapularis* ticks collected by flagging low-level vegetation exhibited widespread distribution in Ontario. Since white-tailed deer and songbirds transport *B. odocoilei* across the Ontario landscape, vertebrate hosts do not need to visit an endemic area to become infected with this babesial piroplasm. 

## Figures and Tables

**Table 1:** Detection of *Babesia odocoilei* in *Ixodes scapularis* adults collected in southern Ontario, 2019.

	Number of *B. odocoilei*-Positive Ticks (%)		
Source	Female(s)	Male(s)	Total Tick(s)
**General sampling**			
Cat	2/4 (50)	0 (0)	2/4 (50)
Dog	13/17 (76.5)	0 (0)	13/17 (76.5)
Vegetation	1/19 (5.3)	3/13 (23.1)	4/32 (12.5)
**Cat and dog sampling**			
Barrie	2/2 (100)	0 (0)	2/2 (100)
East Wasaga	0/1 (0)	0 (0)	0/1 (0)
Orillia	1/2 (50)	0 (0)	1/2 (50)
Oro Medonte	3/3 (100)	0 (0)	3/3 (100)
Penetanguishene	2/3 (66.7)	0 (0)	2/3 (66.7)
Severn Township	1/1 (100)	0 (0)	1/1 (100)
Warmister	1/1 (100)	0 ((0)	1/1 (100)
Wasaga Beach	5/7 (71.4)	0 (0)	5/7 (71.4)
Woods Beach	0/1 (0)	0 (0)	0/1 (0)
**Flagging vegetation**			
Dundas	03 (0)	1/3 (33.3)	1/6 (16.7)
Port Burwell	0/6 (0)	0 (0)	0/6 (0)
Thorold	0/3 (0)	0/3 (0)	0/6 (0)
Turkey Point	0/4 (0)	1/4 (25.0)	1/8 (12.5)
Wainfleet Bog	1/3 (33.3)	1/3 (33.3)	2/6 (33.3)

**Table 2:** Results of DNA sequence analysis and GenBank accession numbers of *Babesia odocoilei* detected in questing and blood-fed *Ixodes scapuaris* ticks in southern Ontario, 2019.

			Date	Sequence	BLAST Results			GenBank
Tick ID	Location	Source	Collected	Length	% of Type Strain	Score	E-Value	Accession no.
CN19-2-2	Dundas	flagging	24 Apr	263	100	521	7e-144	MW182495
CN19-5-2	Wainfleet Bog	flagging	24 Apr	100	100	198	4e-47	MW182496
CN19-6-1	Wainfleet Bog	flagging	24 Apr	163	99.37	307	1e-79	MW182497
CN19-8-2	Turkey Point	flagging	25 Apr	152	100	301	7e-78	MW182498
CN19-79	Penetanguishene	cat	02 May	265	100	525	5e-145	MW182499
CN19-80	Penetanguishene	dog	14 May	212	100	416	2e-112	MW182500
CN19-86	Wasaga Beach	dog	30 Apr	357	100	706	0	MW182501
CN19-87	Wasaga Beach	dog	03 May	361	99.72	708	0	MW182502
CN19-88	Wasaga Beach	dog	09 May	358	99.72	200	0	MW182503
CN19-89	Wasaga Beach	dog	10 May	440	100	872	0	MW182504
CN19-90	Wasaga Beach	dog	10 May	434	100	860	0	MW182505
CN19-91	Severn Township	dog	07 May	440	100	872	0	MW182506
CN19-92	Oro Medonte	dog	14 May	387	100	767	0	MW182507
CN19-93	Barrie	dog	15 May	429	100	848	0	MW182508
CN19-94	Orillia	dog	16 May	429	100	850	0	MW182509
CN19-95	Warmister	dog	17 May	429	100	850	0	MW182510
CN19-96	Oro Medonte	dog	19 May	429	100	850	0	MW182511
CN19-98	Oro Medonte	dog	23 May	429	100	850	0	MW182512
CN19-99	Barrie	cat	24 May	429	100	850	0	MW182513

## Data Availability

Not applicable.
